# DRI-Net: segmentation of polyp in colonoscopy images using dense residual-inception network

**DOI:** 10.3389/fphys.2023.1290820

**Published:** 2023-10-25

**Authors:** Xiaoke Lan, Honghuan Chen, Wenbing Jin

**Affiliations:** College of Internet of Things Technology, Hangzhou Polytechnic, Hangzhou, China

**Keywords:** image segmentation, colonoscopy, residual-inception, dense, down-sampling

## Abstract

Colorectal cancer is a common malignant tumor in the gastrointestinal tract, which usually evolves from adenomatous polyps. However, due to the similarity in color between polyps and their surrounding tissues in colonoscopy images, and their diversity in size, shape, and texture, intelligent diagnosis still remains great challenges. For this reason, we present a novel dense residual-inception network (DRI-Net) which utilizes U-Net as the backbone. Firstly, in order to increase the width of the network, a modified residual-inception block is designed to replace the traditional convolutional, thereby improving its capacity and expressiveness. Moreover, the dense connection scheme is adopted to increase the network depth so that more complex feature inputs can be fitted. Finally, an improved down-sampling module is built to reduce the loss of image feature information. For fair comparison, we validated all method on the Kvasir-SEG dataset using three popular evaluation metrics. Experimental results consistently illustrates that the values of DRI-Net on IoU, Mcc and Dice attain 77.72%, 85.94% and 86.51%, which were 1.41%, 0.66% and 0.75% higher than the suboptimal model. Similarly, through ablation studies, it also demonstrated the effectiveness of our approach in colorectal semantic segmentation.

## 1 Introduction

In today’s world, cancer has become the most important disease threatening human health. Due to genetic, environmental, diet and other factors, there are more and more patients with colorectal cancer, and the death rate is also the second highest. Research shows that colorectal cancer lesions are closely related to colorectal polyps. Therefore, early detection and treatment can effectively control the occurrence of diseases and reduce the mortality rate. By far, colonoscopy is an effective diagnostic method for detecting polyps in the intestine, and it has become the gold standard for early screening of colorectal cancer. Although the size, shape and lesions of tumors can be visually observed through colonoscopy, the characteristic analysis of the pathological images is entirely dependent on the professional doctor. This method not only has a long detection cycle and high labor intensity, but also relies heavily on the subjective judgment and cognition of doctors. Besides, with the increase in the number of disease patients, the demand for professional experts is also increasing, which poses a huge challenge to the medical talent industry. For this reason, the combination of computer vision technology and pathological image diagnosis has become extremely important in the medical field.

At present, deep-learning performs very well in computer vision, especially in medical image-assisted diagnosis (Dang et al., 2023; Maria et al., 2023; Morita et al., 2023; Yang et al., 2023; Zhang et al., 2023). Compared with traditional segmentation frameworks (Srikanth and Bikshalu, 2022; Chen et al., 2023), the core advantage of deep learning is that it can independently discover and learn higher-level image features directly from training data, thus significantly reducing the refinement of feature extraction and facilitating end-to-end image processing in deep architectures. At present, convolutional neural network (CNN) (Lecun et al., 1998) is one of the most popular models in deep learning networks. By introducing local receptive fields, weight sharing, and pooling operations, the generalization ability of the model is greatly improved. However, this network needs to assign labels to each pixel, and medical images often contain millions of pixels, so it takes a lot of time to process millions of forward channels. In addition, all pixels are calculated independently, resulting in spatial inconsistencies in the segmentation results. To solve the above problems, Long et al. (2015) proposed a full convolutional network (FCN). By replacing the fully-connected layer in CNN with a convolutional layer, the spatial information of images can be preserved by using the features and up-sampling strategies of different layers. At the same time, this method can accept any size of input image, and is easier to implement than the traditional image block classification method.

Inspired by FCN, similar network structure models have emerged in an endless stream, mainly improved from extended convolutions (Liu et al., 2020; Karthika and Senthilselvi, 2023), recurrent neural networks (Tan et al., 2021; Chen J et al., 2022), multi-scale features (Dourthe et al., 2022; Goyal et al., 2022), residual connections (Anil and Dayananda, 2023; Selvaraj and Nithiyaraj, 2023) and attention mechanisms (Kanimozhi and Franklin, 2023; Rasti et al., 2023). Among them, Tang et al. (2022) proposed a guidance network for segmentation of medical images that can learn and cope with uncertainty end-to-end. Specifically, this method contains of three parts: firstly, the rough segmentation module is used to obtain the rough segmentation and uncertainty graph. Secondly, the feature refinement module is used to embed multiple double attention blocks to generate the final segmentation. Finally, to extract richer context information, a multi-scale feature extractor is inserted between the encoder and decoder of the coarsely segmented module. Sun et al. (2022) proposed a dual-path CNN with DeepLabV3+ as the backbone. In this method, soft shape monitoring blocks were inserted between the regional path and the shape path to realize the cross-path attention mechanism, so as to accurately detect and segment thyroid nodules. Zhang et al. (2022) proposed a retinal vessel segmentation algorithm based on M-Net. Firstly, to reduce the influence of noise, a double-attention mechanism based on channel and space was designed. Then, the self-attention mechanism in Transformer is introduced into skip connections to recode features and explicitly model remote relationships. Fu et al. (2022) proposed an automatic segmentation method for cardiac MRI images. On the one hand, CNNs were used for feature extraction and spatial encoding of inputs. On the other hand, by using Transformer to add remote dependencies to advanced features, the model’s ability to capture details can be fully utilized.

In this research, we proposed a new dense residual-inception network (called DRI-Net) for the segmentation of colorectal polyps and performed comparative experiments on a public dataset. Compared to other networks, our contributions are the following:

1) Using standard U-Net architecture, the DRI-Net was presented to provide guidance for the accurate segmentation of polyps.
2) In DRI-Net, to make the network structure wider without gradient disappearing, simple convolutional blocks were replaced with dense residual-inception blocks.
3) The down-sampling was carefully redesigned using average-pooling to reduce the loss of image feature information.
4) We do ablation studies on residual-inception, dense and down-sampling. Compared with several classical algorithms, our approach has better performance.

## 2 Methods

**FIGURE 1:**
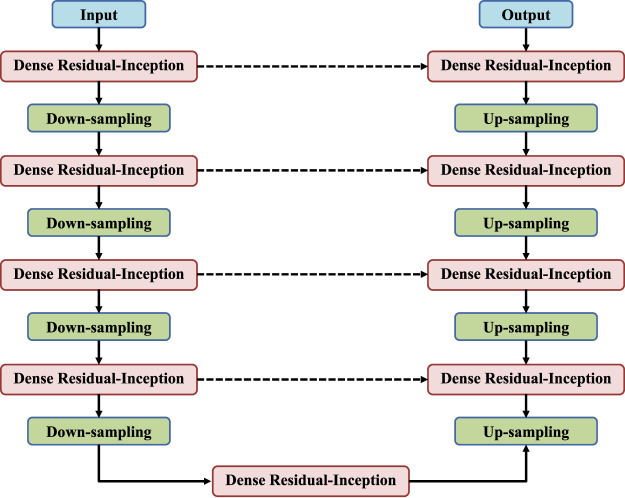
Proposed DRI-Net architecture.

DRI-Net is a classic encoder-decoder structure, and its overall network is shown in Figure 1. The left encoder includes four dense residual-inception modules, and each of which is followed by a pooling layers to down-sample the image. The right decoder also contains four dense residual-inception modules and the resolution is successively increased by the up-sampling operation until it is consistent with the resolution of the input image. Skip connections are used in the network to connect the up-sampled result to the output of a module with the same resolution in the encoder as the input to the next module in the decoder. Finally, the activation function used in the last layer is a Sigmoid function to generate binary segmentation results, and the rest of the activation functions are linear activation functions. In the following, we will explain each block in detail.

### 2.1 Residual-inception

**FIGURE 2:**
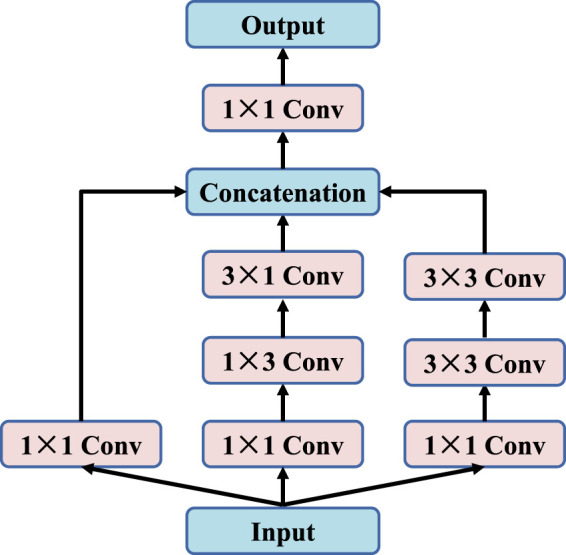
The inception block.

In deep learning, many algorithms achieve better results by simply deepening or broadening neural networks. However, it not only greatly increases the number of parameters and the amount of computation, but also causes problems such as generator over-fitting, gradient disappearing and insufficient diversity of generated samples. To overcome the above difficulties, we propose an improved inception module with multiple convolution kernels of 1 × 1, 1 × 3, 3 × 1 and 3 × 3, as shown in Figure 2. By using parallel structure, the weight of each convolution kernel is adjusted adaptively during the training process, so that the network can adapt to images of different scales. At the same time, three sets of convolution kernels can convert full connection-layer connections to sparse connections, thus improving computational efficiency and extracting more features. However, it is important to note that with the number of convolution cores increases, the number of parameters will increase. Therefore, each group of parallel branches will first undergo 1 × 1 convolution operations to reduce channel dimensions to achieve the purpose of dimensionality reduction of images.

**FIGURE 3:**
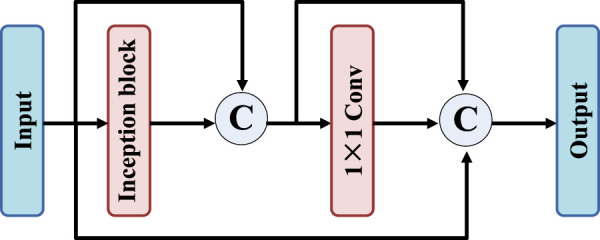
The residual-inception block.

To further improve the feature extraction capability of the network, residual-inception block is designed, as shown in Figure 3. Firstly, the proposed residual-inception structure connects the input and output of inception layer and 1 × 1 convolution layer respectively. This approach presents an overall sequential connection, and the distance between the two connected network layers is short and there is only one network layer. Firstly, the residual-inception structure is proposed to connect the input and output of inception layer and 1 × 1 convolution layer respectively. This approach presents an overall sequential connection, and the distance between the two connected network layers is short and there is only one network layer. Then, the input features of the image are connected with the output of the second connection layer. In this structure, the features of short jump connections include both adjacent outputs and distant ones.

### 2.2 Dense connections

**FIGURE 4:**
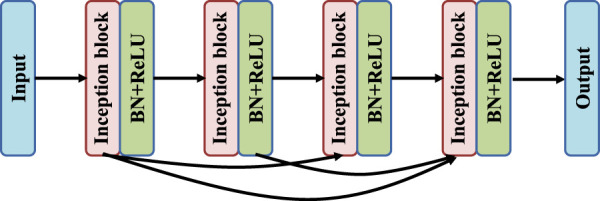
The dense connections block.

As shown in Figure 4, the DRI-Net network adopts a dense structure, and the top convolutional layer is directly connected to the subsequent convolutional layer. After each convolution layer there is a Batch Normalization (BN) layer and a Rectified Linear Unit (ReLU) layer. This connection integrates the larger eigenvalues of the bottom layer into the smaller eigenvalues of the top layer, which can effectively alleviate the problems of over-fitting and gradient disappearance. In addition, the number of existing colon image datasets is small, which will make deep neural network training difficult. At the same time, the disappearance of gradients during training will seriously limit the improvement of the accuracy of neural networks, and dense structures can alleviate these problems to some extent.

### 2.3 Down-sampling layer

**FIGURE 5:**
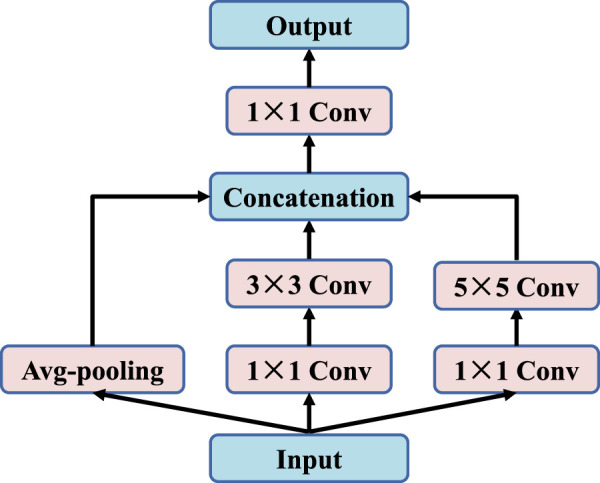
The down-sampling layer.

The traditional U-Net (Ronneberger et al., 2015) uses Max-pooling to reduce and compress features in the shrink path. However, this will cause a lot of useful information in the image to be lost. In order to store more fine-grained feature information and reduce information loss caused by pooling process, this paper adopts two 1 × 1 convolution steps, one 3 × 3 convolution, one 5 × 5 convolution and average-pooling layers for parallel processing, as shown in Figure 5.

## 3 Experiments and results

The colorectal polyp images from Kvasir-SEG (Jha et al., 2020) dataset were used to evaluate the performance of DRI-Net. The database has 1,196 images, of which 700 are training sets, 300 are verification sets, and 196 are test sets. The programming language used in the experiment is Python 3.6, the operating system is Windows 10. The system memory is 24 GB, and the GPU is NVIDIA Quadro RTX 6000. According to the effect of the network, we select the Adam optimizer, the initial learning-rate was 0.001 (Badshah and Ahmad, 2021), the batch size was 16, the number of iterations was 200, the learning rate was 0.001, and the loss function was Dice loss.

### 3.1 Evaluation metrics

Several quantitative metrics, including Intersection over Union (IoU) (Ahmed et al., 2021), Matthews correlation coefficient (Mcc) (Jiang et al., 2021), and Dice (Yang et al., 2020) were adopted to evaluate the performance of each algorithm. The above indicators can calculate as:

$$IoU=\frac{TP}{TP+FN+FP}$$ (1)

$$Mcc=\frac{TP\times TN-FP\times FN}{\sqrt{\left(TP+FN\right)\left(TP+FP\right)\left(TN+FN\right)\left(TN+FP\right)}}$$ (2)

$$DIC=\frac{2TP}{2TP+FN+FP}$$ (3)

where TP indicates that the actual target is a positive sample, and the algorithm also judges the target as a positive sample. TN is represented as a negative sample, and the algorithm also judges this negative sample as a negative sample. FP means a negative sample, but the algorithm incorrectly judges it as a positive sample, FN means a positive sample, but the algorithm incorrectly judges it as a negative sample.

### 3.2 Comparison with other methods

To quantitatively analyze the performance of the models, IoU, Mcc and Dice were calculated for automatic segmentation compared with manual specificity, as shown in Table 1. By adding the gate attention mechanism to the UNet structure, AttUNet, Connected-AttUNet and FF-UNet can effectively improve the precision to segment the colonoscopy images and achieve good results. Among them, the results of DenseUnet, ASF-Net and DRI-Net network are very close, and the comparison between them can objectively reflect the advantages of dense connections. Although the dense mechanism can increase the size of the network and reduce the over-fitting of without using a pre-trained model, its ability to increase the size of the network is limited. Obviously, our approach is superior to other methods in depth feature characterization and can obtain more accurate segmentation results. As you can see from the last two columns, although DRI-Net achieves better segmentation results, it requires more parameters and runtimes due to the introduction of many modules.

**TABLE 1:** The results of comparison with other methods.

	IoU (%)	Mcc (%)	Dice (%)	Parameter (M)	Time (ms/step)
U-Net Ronneberger et al. (2015)	69.09	80.09	81.36	2.06	13
AttUNet Oktay et al. (2018)	73.72	83.13	83.53	8.49	21
DenseUnet Huang et al. (2017)	76.31	85.28	85.76	2.29	17
NestedUNet Zhou et al. (2018)	72.02	82.17	83.13	8.74	33
Connected-AttUNet Baccouche et al. (2021)	0.728825	0.828933	0.836375	5.60	29
ASF-Net Chen P et al. (2022)	0.766183	0.853072	0.862914	5.63	18
FF-UNet Iqbal et al. (2022)	0.7488	0.843762	0.851601	3.97	27
DRI-Net	77.72	85.94	86.51	29.42	102

**FIGURE 6:**
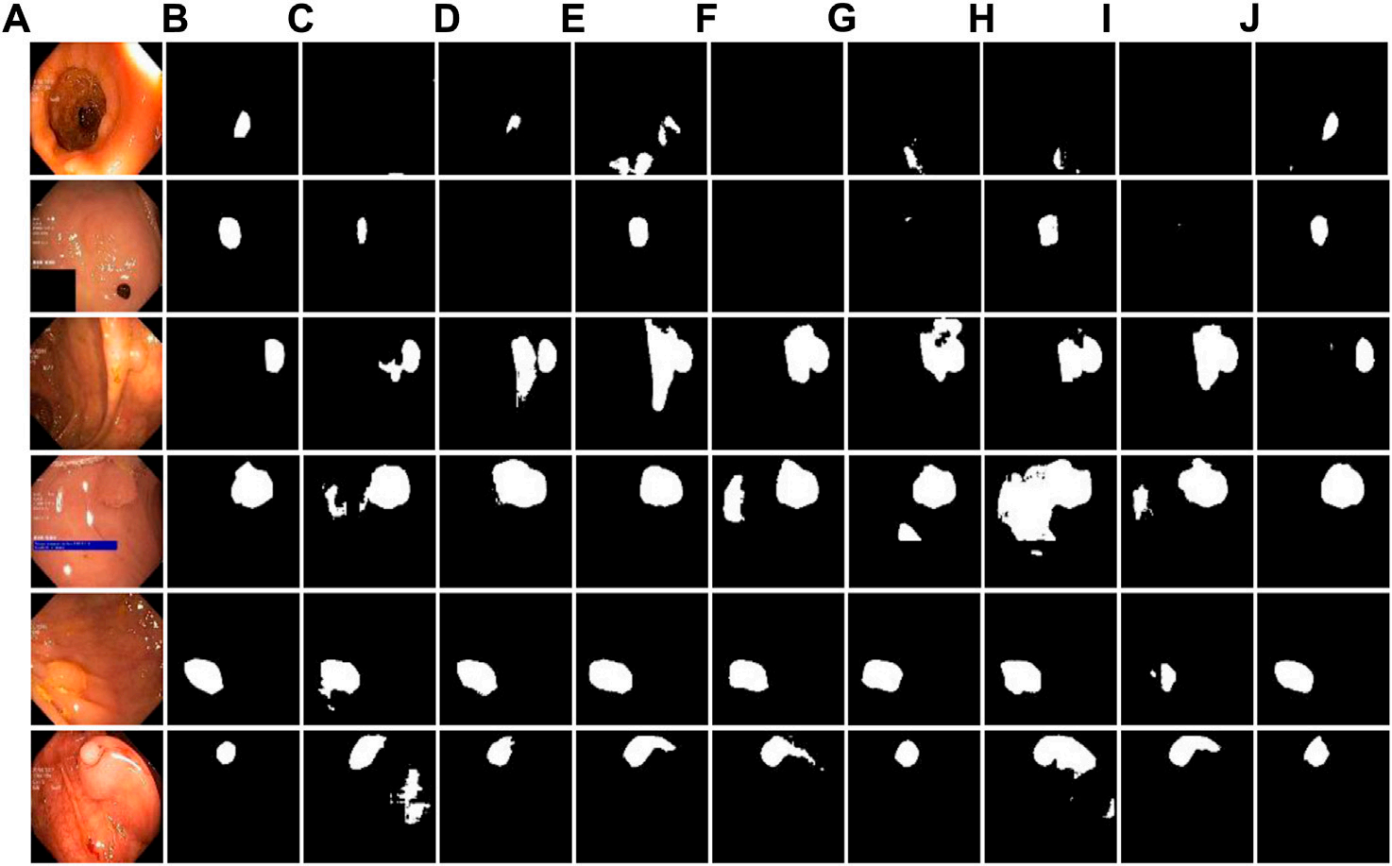
Comparison experiment with other methods on Kvasir-SEG dataset. **(A)** original images; **(B)** Ground-truth; **(C–J)** are the results of U-Net, AttUNet, DenseUnet, NestedUNet, Connected-AttUNet, ASF-Net, FF-UNet and DRI-Net.

Figure 6 shows the comparison of the visualization segmentation results on the Kvasir-SEG dataset between the DRI-Net and the models proposed by some researchers in recent years. It can be seen from the segmentation example that in the structure of U-net, due to the lack of support for convolutional low-level information, the segmentation details are poor and there are many false negatives. Compared with U-Net, the results of other models are better, and the false negative is reduced. However, due to the loss of global association, the phenomenon of over-segmentation appeared, and the false positives of polyp segmentation were relatively high. As can be seen from the comparison between the visual segmentation results and Ground-truth, compared with other methods, our method can well distinguish polyp boundaries, and is better in maintaining the consistency of polyp morphological features, with lower FP and FN.

In order to prove that each module added to the proposed DRI-Net network plays a role in colorectal polyp images, ablation experiments are conducted for each module. Experimental comparisons were conducted on the Kvasir-SEG dataset using U-Net, U-Net with only residual-inception module, U-Net with only dense module, U-Net with only down-sampling module, U-Net with residual-inception+dense module, U-Net with residual-inception+down-sampling module, U-Net model with dense+down-sampling module, and DRI-Net. As can be seen from Table 2, when the network is added with the combination of residual-inception, dense and down-sampling modules, compared with a single U-Net, all evaluation indicators are better than those obtained by the latter, it demonstrate the effectiveness of the these modules.

**TABLE 2:** Ablation experiment of DRI-Net on Kvasir-SEG dataset.

U-net	Residual-inception	Dense	Down-sampling	IoU (%)	Mcc (%)	Dice (%)
**√**	**×**	**×**	**×**	69.09	80.09	81.36
**√**	**√**	**×**	**×**	73.27	83.17	84.16
**√**	**×**	**√**	**×**	74.67	83.95	84.70
**√**	**×**	**×**	**√**	72.45	82.64	83.26
**√**	**√**	**√**	**×**	75.77	84.93	85.61
**√**	**√**	**×**	**√**	75.28	84.75	85.42
**√**	**×**	**√**	**√**	75.61	84.76	85.38
**√**	**√**	**√**	**√**	77.72	85.94	86.51

**FIGURE 7:**
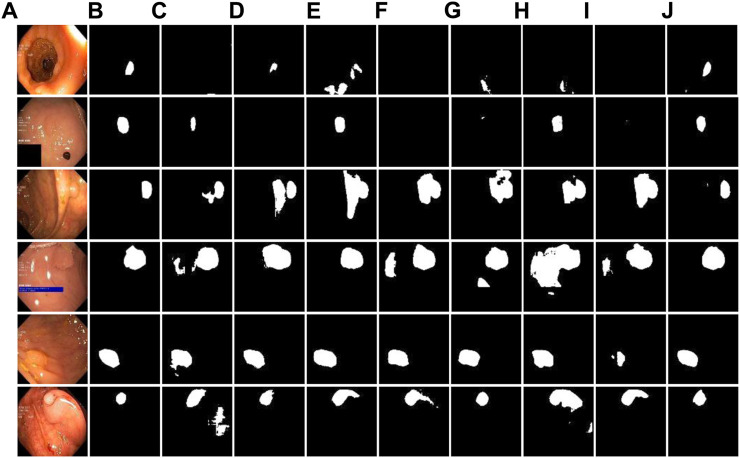
Ablation experiment of DRI-Net on Kvasir-SEG dataset. **(A)** original images; **(B)** Ground-truth; **(C–J)** are the combination results of baseline (U-Net), U-Net+residual-inception, U-Net+dense, U-Net+down-sampling, U-Net+residual-inception+dense, U-Net+residual-inception+down-sampling, U-Net+dense+down-sampling, and U-Net+residual-inception+dense+down-sampling.

For further analysis of ablation performance, the partial segmentation results are shown in Figure 7. Visually, it can be seen that before module fusion, there was still under segmentation at the boundaries of some lesions, and the complete tumor region could not be segmented well. After adding residual-inception, dense and improved down-sampling modules, the segmentation accuracy of the whole network is greatly contributed. Therefore, the results of ablation experiments further verify the validity of these modules.

## 4 Conclusion

Based on the color similarity between colon polyps and surrounding tissues and the diversity of size, shape and texture of colon polyps, a dense residual initialization network structure is proposed, which is an effective extension of encoder-decoder U-Net network. Firstly, we integrate the reside-inception module and dense connection into U-Net to effectively extract more discernible features in colon cancer tissue from a large amount of information. Then, the re-designed down-sampling module aim to suppress useless information and improve the recognition accuracy of the network. We assessed all methods on the Kvasir-SEG dataset using three popular evaluation metrics. Experimental results consistently illustrates that DRI-Net has better results than other typical networks. In the future, we will investigate the lightweight and over-fitting problems of these methods and apply them to more medical image segmentation tasks.

## Data Availability

The original contributions presented in the study are included in the article/Supplementary Material, further inquiries can be directed to the corresponding author.
